# A Systematic Review of Workplace Interventions to Rehabilitate Musculoskeletal Disorders Among Employees with Physical Demanding Work

**DOI:** 10.1007/s10926-020-09879-x

**Published:** 2020-03-26

**Authors:** Emil Sundstrup, Karina Glies Vincents Seeberg, Elizabeth Bengtsen, Lars Louis Andersen

**Affiliations:** 1 National Research Centre for the Working Environment (NRCWE), Lersø Parkallé 105, 2100 Copenhagen Ø, Denmark; 2 Sport Sciences, Department of Health Science and Technology, Aalborg University, 9220 Aalborg, Denmark

**Keywords:** Occupational health, Pain, Physical demands, Physical exercise, Strength training, Participatory ergonomics, Ergonomics, Stress management

## Abstract

*Purpose* This systematic review investigates the effectiveness of workplace interventions to rehabilitate musculoskeletal disorders (MSDs) among employees with physically demanding work. *Methods* A systematic search was conducted in bibliographic databases including PubMed and Web of Science Core Collection for English articles published from 1998 to 2018. The PICO strategy guided the assessment of study relevance and the bibliographical search for randomized controlled trials (RCTs) and non-RCTs in which (1) participants were adult workers with physically demanding work and MSD (including specific and non-specific MSD and musculoskeletal pain, symptoms, and discomfort), (2) interventions were initiated and/or carried out at the workplace, (3) a comparison group was included, and (4) a measure of MSD was reported (including musculoskeletal pain, symptoms, prevalence or discomfort). The quality assessment and evidence synthesis adhered to the guidelines developed by the Institute for Work & Health (Toronto, Canada) focusing on developing practical recommendations for stakeholders. Relevant stakeholders were engaged in the review process. *Results *Level of evidence from 54 high and medium quality studies showed moderate evidence of a positive effect of physical exercise. Within this domain, there was strong evidence of a positive effect of workplace strength training. There was limited evidence for ergonomics and strong evidence for no benefit of participatory ergonomics, multifaceted interventions, and stress management. No intervention domains were associated with “negative effects”. *Conclusions* The evidence synthesis recommends that implementing strength training at the workplace can reduce MSD among workers with physically demanding work. In regard to workplace ergonomics, there was not enough evidence from the scientific literature to guide current practices. Based on the scientific literature, participatory ergonomics and multifaceted workplace interventions seem to have no beneficial effect on reducing MSD among this group of workers. As these interventional domains were very heterogeneous, it should also be recognized that general conclusions about their effectiveness should be done with care.

*Systematic review registration* PROSPERO CRD42018116752 (https://www.crd.york.ac.uk/prospero/display_record.php?RecordID=116752).

## Introduction

Although there has been a major focus on rehabilitating musculoskeletal disorders (MSDs), it remains a significant problem in many workplaces around the world. Data from the Working Environment and Health study, representing the general working population in Denmark, show that the proportion with musculoskeletal pain several times a week has increased from 31% in 2012 to 33% in 2018 [1]. Specifically, low back and neck pain is highly prevalent among workers and the leading causes of disability in high-income countries [2]. At a global level, disability caused by low back pain has increased by more than 50% since 1990 [3]. MSD has a multifactorial etiology and, in addition to individual factors, is influenced by a complex interaction between both physical and psychosocial factors in the working environment [4–7]. MSDs are especially a major problem among workers with physically demanding work (i.e. certain physical tasks are required to perform the job e.g. lifting, pulling, pushing, standing, walking, bending, forceful or fast repetitive tasks, etc.), where pain can make it difficult to perform daily work tasks. Importantly, physical demands at work play an important role in both developing and sustaining MSD. While some are able to work with an MSD, it can for others lead to an imbalance between physical demands of work and individual resources consequently increasing the risk of poor work ability, sick leave and premature exit from the labour market [8–11].

Risk factors and effective solutions for MSDs vary from industry to industry (i.e. group of companies or workplaces that are related based on their primary business activities), and especially between workplaces constituting physical or sedentary labour. Therefore, it is recommended that individual workplaces address the risk factors that are most important to them and chose solutions applicable to their work context [12]. Compared to sedentary workplaces (i.e. office-work), it may also be more challenging to successfully implement effective solutions in workplaces with physically demanding work due to the obvious differences in both the nature of work, workstation design and work organization [13]. Additionally, the workplaces motivation for employing evidence-based research in practice is higher the more specific and tailored the recommendations are. Thus, general advice about reducing MSDs at the workplace can be difficult to translate into practice by the Occupational Health and Safety practitioners (OHS). Practitioners, therefore, request evidence-based approaches to better identify and implement effective interventions for employees with physically demanding work. Such evidence-based knowledge will give relevant practitioners (e.g. OSH practitioners) a stronger knowledge base to act on and may suit them better to choose the best solution applicable to their context of work.

Currently, there are no known published systematic reviews documenting and summarising the literature on the effect of workplace-based interventions specifically for workers with MSD and physically demanding employment. Previous systematic reviews within this topic have mainly focused on MSD in one body region among either the general working population (including both physically demanding and sedentary employment) or among a specific job group (such as health care workers or office workers). A systematic review by Van Hoof et al. [14] only found four relevant randomized controlled trials (RCTs) with low risk of bias and concluded that there is no strong evidence for any intervention in treating or preventing low back pain in nurses. Further, Verbeek et al. [15] found no available evidence from RCTs for the effectiveness of manual material handling advice and training or manual material handling assistive devices for treating back pain. Thus, they concluded that more high-quality studies could further reduce the remaining uncertainty. A systematic review by Skamagki et al. [16] found that workplace interventions such as high‐intensity strength exercises and/or integrated health care can decrease pain and symptoms for employees who experience long‐term musculoskeletal disorders. Overall, these reviews base their evidence synthesis on RCTs, and all concluded that current research is limited. Even though RCTs are considered the most powerful experimental design in clinical trials, solely including these may be too restrictive to understand effective workplace-based interventions where randomized and carefully controlled trials (RCTs) are not always possible. Furthermore, a high-quality RCT does not guarantee that a workplace intervention has been implemented in a good manner.

Previous reviews have dealt with this methodological challenge by including both RCTs and non-RCTs. To further increase the relevance for practice, these reviews have also employed the quality assessment and evidence synthesis developed by the Institute for Work & Health (IWH, Toronto, Canada) which focuses on the development of practical guidelines for stakeholders. In such a review process, Van Eerd et al. [13] investigated the effectiveness of workplace interventions in the prevention of upper extremity MSDs and symptoms. They found strong evidence for the intervention category resistance training (one among 30 categories), leading to the following recommendation for stakeholders: “Implementing a workplace-based resistance training exercise program can help prevent and manage upper extremity MSDs and symptoms” [13]. The review also reported moderate evidence for the effect of stretching, mouse use feedback and forearm supports and moderate evidence for no effect of EMG biofeedback, job stress management training, and office workstation adjustment. Further, Hossain et al. (2019) investigated the evidence on the effectiveness of workplace-based rehabilitative interventions in workers with upper-limb conditions also by including RCTs and non-RCTs along with the review process developed by the IWH [17]. They found that the largest body of evidence supported workplace physical exercise programs, but also reported positive effects for ergonomic training and workstation adjustments, and mixed-effects for ergonomic controls.

The aim of this systematic review is to investigate the effectiveness of workplace interventions to rehabilitate musculoskeletal disorders among employees with physically demanding work. Workplace interventions are here defined as interventions that are initiated by the workplace, supported by the workplace, and/or carried out at the workplace. Level of evidence will be synthesized within several broad intervention domains such as physical exercise, ergonomics, participatory ergonomics, and multifaceted interventions. If possible, due to a data-driven approach, each domain will further be divided into more specific intervention categories. Based on the evidence synthesis, practical messages for stakeholders will be developed. To introduce a more practical approach, relevant stakeholders are engaged in the review process and both RCTs and non-RCTs are eligible for inclusion.

## Methods

### Study Design and Registration

This systematic review followed the ‘Preferred Reporting Items for Systematic reviews and Meta-Analyses’ (PRISMA) guidelines for reporting systematic reviews and the IWH guideline for workplace-based interventions. Inspired by the IWH Systematic Review Programme [18], relevant stakeholders were engaged in parts of the review process. The stakeholders were members of two industry communities for work environment representing workers with physically demanding work within construction and manufacturing. The communities, which consist of relevant representatives from both employers', managers' and employees' labour market organizations, support the workplaces with information and guidance on the working environment by, among other things, making guidelines, conferences and education. To ensure maximal practical relevance of the present work, the stakeholders were involved in the conception of the study through involvement in the preparation of the research application. This ensured that the topic was practical and relevant to our stakeholders. When funding for the study was obtained, the stakeholders participated in a meeting to discuss and finalize the research question, and they provided practical input to the search strategy. This helped to ensure that the literature search was comprehensive. At the meeting, the researchers also gave the stakeholders a short introduction to the systematic review steps and evidence synthesis methodology. This was done to increase the research capacity of the engaged stakeholders and to prepare them to better understand, interpret and disseminate the results of the review. After the forming of the evidence synthesis, the results were presented for the stakeholders and they provided input to the recommendations and the dissemination of the results.

The review has been registered in the International Prospective Register of Systematic Reviews (PROSPERO) number CRD42018116752 and a protocol paper has previously been published [19].

### Eligibility Criteria

Eligibility criteria can be seen in Table 1 illustrating the PICO employed for the present review. The PICO strategy guided the assessment of study relevance and the bibliographical search for studies in which (1) participants were adult workers with physically demanding work and MSD (including specific and non-specific MSD and musculoskeletal pain, symptoms, and discomfort), (2) interventions were initiated and/or carried out at the workplace, (3) a comparison group was included, and (4) a measure of MSD was reported (including musculoskeletal pain, symptoms, prevalence or discomfort). In addition, both RCTs and non-RCTs were included and the publication language of included studies was English. If it was not possible to identify whether individual studies were workplace-based or not, authors were contacted for clarification. Physically demanding work was defined as work that is physically demanding on a whole-body level or for specific body parts and where certain physical tasks are required to perform the job (e.g. lifting, pulling, pushing, standing, walking, bending, forceful repetitive tasks, etc.). Thus, industries with mainly physical demanding work—such as construction work, automotive work, health care work, slaughterhouse work etc.—were included in the review. If no specific industry was reported in the paper, it should have been specifically stated that the participants were engaged in physically demanding work or rated their work as being physically demanding. Studies where it was not possible to identify whether participants had physically demanding work, or where the participants constituted a mix of workers with sedentary and physical work (without a stratified effect evaluation) were not included in the review.

**Table 1:** Illustration of the PICO used for the present review

P	Population	Adult workers with physically demanding work and MSD (including specific and non-specific MSD and musculoskeletal pain, symptoms, and discomfort)
I	Intervention	The intervention was initiated by the workplace, supported by the workplace and/or carried out at the workplace (i.e. workplace-based)
C	Comparison	A comparison group was included (i.e. no treatment, treatment as usual, or another comparison treatment at the workplace)
O	Outcome	Effective in decreasing a measure of MSD (including musculoskeletal pain, symptoms, prevalence or discomfort)

### Search Strategy

The systematic search was conducted in the following bibliographic databases: PubMed (including the database ‘MEDLINE’) and Web of Science Core Collection (including the databases ‘Science Citation Index Expanded’, ‘Social Sciences Citation Index’ and ‘Arts & Humanities Citation Index’). The search strategy consisted of combining the following four main components: (1) musculoskeletal diseases/disorder AND (2) workers AND (3) workplace intervention AND (4) date (published within the last 20 years: from 1998 to 2018). The search strategy for each database has previously been reported [19]. Manual searches were also performed by employing the ‘Snowball’ method. Specifically, we pursued references of paramount references within the field of MSD prevention at the workplace. Importantly, the ‘Snowball’ method did not provide any additional papers to the study after the search was conducted, but was used to optimize the search strategy, making it more agile to identify pre-defined key papers for this review. In addition, relevant articles identified through personal knowledge and contacts were also included in the review process (25).

### Assessment of Relevance and Inclusion

**Fig. 1:**
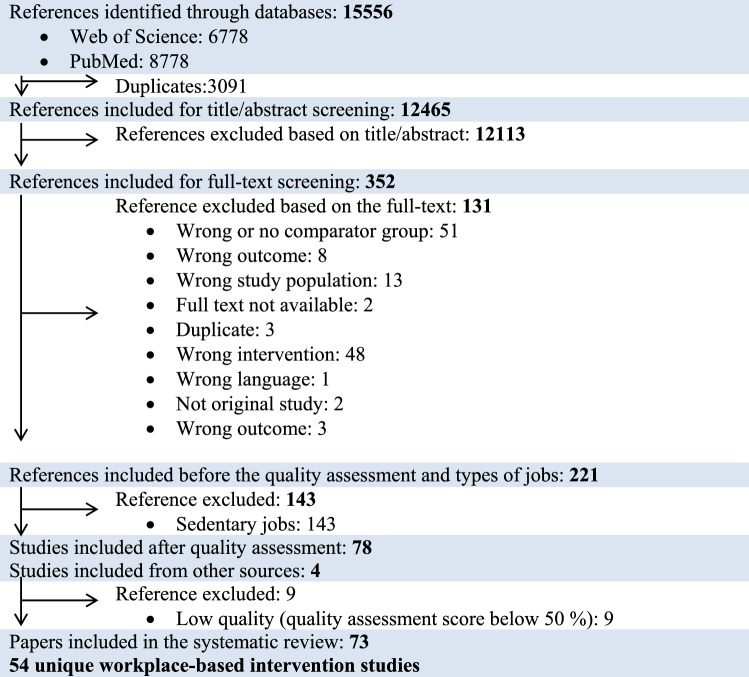
Flow chart

The PRISMA flow-diagram illustrated in Fig. 1 summarizes the study selection process. EndNote X8 was employed to collect all potential studies from PubMed and Web of Science Core Collection. The selected studies were exported to the review software program Covidence. Abstracts of potential studies were thereafter independently assessed by the first author (ES) and the coauthor (KGVS). Any disagreements were discussed with the senior author (LLA) until a consensus was achieved. Full-text publications of those studies deemed relevant by the abstract screening were thereafter assessed in a similar manner. The studies, which adhered to the eligibility criteria presented in the PICO (Table 1), were included in the systematic review. Since the effectiveness of interventions for workers with sedentary employment will be reported in a separate paper, studies involving sedentary workers were excluded in a separate step during the full-text screening (Fig. 1). Thereafter, studies were assessed for quality and the best evidence synthesis was formed. Only high and medium quality studies were eligible for the evidence synthesis, whereas studies with low quality were not sufficient to move forward to data extraction.

### Data Extraction

For each included study, systematic data extraction was employed to collect the following general characteristics: (1) author year and country, (2) study design, (3) study population, (4) intervention and comparison, (5) number of participants, (6) time-frames of outcome measurement, (7) results, and (8) quality appraisal.

Since MSDs are a diverse group of conditions, several different outcome measures have been employed in the literature. Thus, we decided not to exclude potential relevant studies due to heterogeneity in outcomes, as long as they represented a measure related to MSD. The outcomes employed for the quality appraisal and evidence synthesis, therefore, included any change in musculoskeletal pain, symptoms, prevalence or discomfort from baseline to follow-up (see the PICO illustrated in Table 1). Thus, outcomes employed were not only primary outcome measures, but could also reflect secondary or tertiary measures. If several follow-up periods were reported, data from the longest follow-up time-period was employed for the evidence synthesis (unless specifically stated in the study aim that a given follow-up time was the primary focus of the study). If a study reported on several MSD outcome-measures (for instance pain in many different body regions) the outcome of interest for the present review was the one that was predefined as the primary MSD-related outcome in the aim/methods. If no such definition was provided, the outcome measure for the evidence synthesis adhered to the body-region with the highest intensity or frequency of MSD (pain, symptoms or discomfort) or the region with the highest prevalence of MSD at baseline (if intensity or frequency was not reported).

### Assessment of Quality

Two authors (ES and KVGS) independently assessed the quality of each study and any disagreements were discussed with the senior author (LLA) until a consensus was reached. For the methodological appraisal, we used the quality assessment methods developed by the IWH consisting of 16 unique questions (see Table 2). The IWH quality assessment score for each article was based on a weighted sum score [13, 20]. The weighting values of each question ranged from 1 to 3. The rank score for each included study was divided by the maximal weighted sum score and multiplied by 100. Finally, the studies were divided into three groups depending on the ranking score: low quality (below 50%), medium quality (50–85%) and high quality (> 85%) [20, 21]. Only high and medium quality studies were eligible for the evidence synthesis [20, 22].

**Table 2:** Assessing methodological quality ([20] adapted from Kennedy et al. 2010

Question	Weight
1. Is the research question clearly stated?	2
2. Were comparison group(s) used?	3
3. Was an intervention allocation described adequately? (and was it randomized?)	3*2
4. Was recruitment (or participation) rate reported?	2
5. Were pre-intervention characteristics described?	2
6. Was loss to follow-up (attrition) < 35%?	2
7. Did the author examine for important differences between the remaining and drop-out participants after the intervention?	2
8. Was the intervention process adequately described to allow for replication?	3
9. Were the effects of the intervention on some exposure parameters documented?	1
10. Was the participation in the intervention documented?	2
11. Were musculoskeletal pain, symptoms, discomfort and/or disorders described at baseline and at follow-up	3
12. Was the length of follow-up three months or greater?	2
13. Was there adjustment for pre-intervention differences (minimum threshold of three important covariates include age, gender and primary outcome at baseline)?	3
14. Were the statistical analyses optimized for the best results?	3
15. Were all participants’ outcomes analyzed by the groups to which they were originally allocated (intention-to-treat analysis)?	2
16. Was there a direct between-group comparison?	3

### Assessment of Evidence

We employed the IWH adapted “best evidence synthesis approach” to clarify the evidence (see Table 3). The approach considers the article’s quality, the quantity of articles evaluating the same intervention and finding consistency [20, 22]. Based on this, the level of evidence was classified as ‘strong’, ‘moderate’, ‘limited’, ‘mixed’ or ‘insufficient’ based on the quality assessment of the included studies. A strong level of evidence resulted in “recommendations” for practice and a moderate level of evidence resulted in “practice recommendations” or practices to be considered for workplace management of MSD [20, 22]. An evidence level below moderate (i.e. limited, mixed or insufficient) led to the following message for practice: “Not enough evidence from the scientific literature to guide current policies/practices” [23]. Importantly, this does not mean that the interventions may not be effective, but there is not enough scientific evidence to extract conclusions [23].

**Table 3:** Best evidence synthesis guidelines ([20] adapted from Kenney et al. 2010

Level of evidence	Minimum quality	Minimum quantity	Consistency	Terminology for messages
Strong	High (> 85%)	Three	Three high quality studies agree If more than three studies, 3/4th of the medium and high quality studies agree	Recommendations
Moderate	Medium (50–85%)	Two high quality OR Two medium quality and one high quality	Two high quality studies agree OR Two medium quality studies and one high quality study agree. If more than three studies, more than 2/3rd of the medium and high quality studies agree	Practice considerations
Limited	Medium (50–85%)	One high quality OR Two medium quality OR One medium quality and one high quality	If two studies (medium and/or high quality), agree If more than two studies, more then 1/2 of the medium and high quality studies agree	
Mixed	Medium and high	Two	Findings from medium and high quality studies are contradictory	
Insufficient	No high quality studies, only one medium quality study, and/or any number of low quality studies			

To reach a strong level of evidence, a minimum of three high-quality studies had to point in the same direction (i.e. all showing either positive, negative or no effect of the given intervention), or at least ¾ of all the studies within a specific intervention category or domain (explained below) had to have the same direction of effect [23, 23]. This is illustrated in the best evidence synthesis in Table 3.

Level of evidence was synthesized for high and medium quality studies within the following 5 broad intervention domains agreed on by the authors: physical exercise, ergonomics, participatory ergonomics, stress management, and multifaceted. If deemed possible by the authors, due to both a practical and a data-driven approach, each domain was further divided into relevant intervention categories. For instance, the domain of physical exercise was divided into strength training at the workplace, aerobic training at the workplace, and stretching at the workplace. This allowed for a more specific and stakeholder-friendly evidence synthesis. When consensus was reached by the review team on the intervention domains and categories, evidence was synthesized for each domain and category.

## Results

### Study Selection

The bibliographic searches identified 15,556 articles, of which 3091 were duplicates. Of the 12,465 remaining articles, 12,113 were excluded in the abstract screening as they did not meet our eligibility criteria. Of the 352 full-text articles retrieved, 221 met the overall inclusion criteria. Since the aim of this review was to investigate the effectiveness of interventions for workers with physically demanding work, we only included the articles describing this population (n = 78). The remaining articles (143) will be reported in a separate paper, reporting the effectiveness of interventions for workers with sedentary employment.

In addition to the 78 articles identified for this review, 4 articles were sent to the review team by colleagues, which were published after the systematic search was carried out. In, total 82 papers were included in the review. 19 of these 82 articles reported different outcome measures (i.e. primary or secondary outcome measures not related to MSD) of a unique interventional study (see flow chart; Fig. 1) and were therefore not included in the evidence synthesis. In total, 54 studies were included in the evidence synthesis. Table 4 summarizes the characteristics of the included studies: (1) author year and country, (2) study design, (3) study population, (4) intervention and comparison, (5) number of participants (n), (6) time-frames of outcome measurement (follow-up), (7) results (interventional effect and region of MSD), and (8) quality appraisal.

**Table 4:** Characteristics of the included studies grouped within the 5 overall intervention domains: physical exercise, ergonomics, participatory ergonomics, multifaceted and stress management. Three interventional categories were further established within the domain of physical exercise: strength training, aerobic training, stretching. Four studies did not match any of the overall intervention domains and are presented as “Other” interventions. The characteristics include (1) author year and country, (2) study design, (3) study population, (4) intervention and comparison, (5) number of participants (n), (6) time-frames of outcome measurement (follow-up), (7) results (interventional effect and region of MSD), and (8) quality appraisal (H = high quality, M = medium quality)

Author, year, country	Study design	Study population	Intervention and comparison	n	Follow-up	Results (effect and region of MSD)	QA
**Physical exercise**							
**Strength training**							
Jakobsen (2015) Denmark [24]	RCT	Healthcare workers	I = High-intensity strength training and coaching	111	10 weeks	Yes (p ≤ 0.0003) Low back and neck/shoulder	H
			C = Control (home-based exercises)	89			
Rasotto (2015) Italy [25]	RCT	Manufacturing workers	I = Mobilization and strength training	30	6 months	Yes (p = 0.039) Shoulder	H
			C = Control (no intervention)	30			
Sundstrup (2014) Denmark [26]	RCT	Slaughterhouse workers	I = High-intensity strength training	33	10 weeks	Yes (p < 0.0001) Shoulder, arm and hand	H
			C = Control (individualized ergonomic training and education)	33			
Zebis (2011) Denmark [27]	RCT	Laboratory technicians	I = High-intensity strength training	282	20 weeks	Yes (p < 0.001, p = 0.07) Neck and shoulder	H
			C = Control (received advice to stay physically active)	255			
Munoz-poblete (2019) Chile [28]	RCT	Manufacturing workers	I = Strength training with progressive resistance	52	16 weeks	Yes (p = 0.007, p = 0.045, p = 0.259 p = 0.481, p = 0.016, p = 0.182, p = 0.034, p = 0.013) Upper limb, neck, right and left shoulders, right and left elbows, right and left wrists	H
			C = Control (stretching exercise)	53			
Jay (2011) Denmark [30]	RCT	Laboratory technicians	I = High-intensity strength training	20	8 weeks	Yes (p = 0.02, p = 0.05) Neck/shoulder, low back	M
			C = Control (received recommendation to continue their usual physical activities)	20			
Rasotto (2015) Italy [32]	RCT	Metal workers	I = Mobilization and strength training	34	5 months	Yes (p = 0.0043, p = 0.1037, p = 0.2053, p = 0.0080) Neck, shoulder, elbow and wrist	M
					10 months	Yes (p = 0.0164, p = 0.0224, p = 0.3429, p = 0.0007) Neck, shoulder, elbow and wrist	
			C = Control (continue in performing their normal daily activities)	34			
Balaguier (2017) France [29]	Non-RCT	Vineyard workers	I = Morning: warm-up. After work: trunk flexor, extensor strengthening or/and trunk stretching	10	4 weeks	No (p > 0.05) Low back	M
					8 weeks	Yes (p < 0.01) Low back	
					12 weeks	Yes (p < 0.05) Low back	
			C = Control (not further described)	7			
Oldervoll (2001) Norway [31]	Non-RCT	Hospital employees	I:2 = Strength training	24	15 weeks	Yes (p = 0.031) Neck, shoulder, and lower back	M
			C = Control (continue their normal daily activities)	19			
**Aerobic training**							
Korshoj (2018) Denmark [33]	RCT	Cleaners	I = Aerobic exercise	57	4 months	No (p = 0.80) Low back	H
			C = Control (lectures on healthy living only)	59	12 months	No (p = 0.72) Low back	
Barene (2014) Norway [34]	RCT	Hospital employees	I:2 = Soccer	37	12 weeks	Yes (p = 0.001) Neck and shoulder No (p > 0.05) Lower back region	H
					40 weeks	Yes (p = 0.002) Neck and shoulder No (p > 0.05) Lower back region	
			C = Control (not further described)	35			
Eriksen (2002) Norway [35]	RCT	Postal workers	I:3 = Physical aerobic exercise	189	12 weeks	No (p = 0.517) Head, neck, upper back and low back, arm, shoulder, and leg	H
			C = Control (not further described)	344			
Horneij (2001) Sweden [36]	RCT	Homecare workers	I:2 = Physical exercise (aerobic and stretching)	90	12 months	Yes (p = 0.02) Low back	H
			C = Control (usual care)	93			
Oldervoll (2001) Norway [31]	Non-RCT	Hospital employees	I:1 = Aerobic exercise	22	15 weeks	Yes (p = 0.031) Neck, shoulder, and low back	M
			C = Control (continue their normal daily activities)	19			
**Stretching**							
Bertozzi (2015) Italy [37]	Non-RCT	Poultry slaughterhouse workers	I = Bodyweight and postural exercises, relaxation, stretching, and extension	20	5 weeks	No (p = 0.7, p = 0.1) Cervical and lumber	H
			C = Home exercise	20			
Holmstrom (2005) Sweden [38]	Non-RCT	Construction workers	I = Morning warm-up including stretching exercises	37	3 months	No (p > 0.05) Back	M
			C = Control (not further described)	20			
Han (2016) Korea [39]	Non-RCT	Automotive (assembly line)	I:1 = Pelvic control hamstring stretch	34	6 weeks	Yes (p < 0.05) Low back	M
			I:2 = General hamstring stretch	34	6 weeks	Yes (p < 0.05) Low back	
			C = Control (home stretching)	32			
**General physical exercise**							
Andersen (2015) Denmark [40]	RCT	Healthcare workers	I = Aerobic fitness and strength training	27	3 months	Yes (p ≤ 0.01) All body parts/regions	H
			C = Control (received health guidance only)	27			
Barene (2014) Norway [34]	RCT	Hospital employees	I:1 = Zumba	35	12 weeks	Yes (p = 0.01) Neck and shoulder	H
					40 weeks	No (p = 0.13) Neck and shoulder	
			C = Control (not further described)	35			
Gram (2012) Denmark [41]	RCT	Constructions workers	I = Aerobic exercise and strength training	35	12 weeks	No (p = 0.96, p = 0.11, p = 0.37, p = 0.31, p = 0.92, p = 0.73, p = 0.74, p = 0.70) Neck, shoulder: right, left and dominant, upper back, low back, hip, knee	H
			C = Control (given 1-hour lecture on general health promotion)	32			
Jorgensen (2011) Denmark [42]	RCT	Cleaners	I:1 = Physical coordination training	95	12 months	No (p > 0.05) Neck, shoulder and low back	H
			C = Control (healthcare check, pulmonary test and aerobic capacity test)	100			
Burger (2012) Switzerland [43]	Non-RCT	Manufacturing workers	I = Whole-body vibration training	22	4 weeks	Yes (p < 0.01) All body parts/regions	M
			C = Control (no treatment)	16			
**Ergonomics**							
Jensen (2006) Denmark [45]	RCT	Healthcare workers	I:1 = Ergonomics; practical class room education/instruction	61	3 months	No (p = 0.16) Low back	H
					12 months	No (p = 0.10) Low back	
			C = Control (lessons on skincare, proper treatment of persons with diabetes, and asthma and safety procedures in chemicals handling)	49			
Oleske (2007) USA [46]	RCT	Industrial workers (automotive)	I:1 = Back support + education	222	12 months	No (p = 0.091) Low back	H
			C = Control (education)	211			
Roelofs (2007) Netherlands [47]	RCT	Healthcare workers	I = Lumbar support	183	12 months	Yes (p = 0.020) Low back	H
			C = Control (not further described)	177			
Warming (2008) Denmark [49]	RCT	Nurses	I:1 = Ergonomics; transfer technique instruction, physical exercise	50	12 months	No (p > 0.05) Low back	H
			I:2 = Transfer technique instruction	55	12 months	No (p > 0.05) Low back	
			C = Control (usual care)	76			
Hagiwara (2017) Japan [50]	RCT	Healthcare workers	I = Lumbar support	59	3 months	Yes (p = 0.036) Knee, shoulder, neck, back	M
			C = Control (no intervention)	60			
Yassi (2001) Canada [56]	RCT	Healthcare workers	I:1 = Safe lifting program	116	6 months	No (p > 0.05) Low back and shoulder	M
					12 months	Yes (p = 0.009, p = 0.041) Low back and shoulder	
			I:2 = No strenuous lifting program	127	6 months	Yes (p = 0.015, p = 0.037) Low back and shoulder	
					12 months	No (p > 0.05) Low back and shoulder	
			C = Control (usual practice)	103			
Shojaei (2017) Iran [48]	Non-RCT	Nurses	I = Educational program and ergonomic posture training	63	6 months	Yes (p < 0.001) Low back	H
			C = Control (no intervention)	62			
Hartvigsen (2005) Denmark [44]	Non-RCT	Nurses	I = Educated in body mechanics, patient transfer, and lifting techniques, and use of low-tech ergonomic aids	171	24 months	No (p < 0.88) Low back	H
			C = Control (instruction in lifting technique)	145			
Iwakiri (2018) Japan [51]	Non-RCT	Care workers	I = Ergonomic education program	49	12 months	No (p = 0.69) Low back	M
					18 months	No (p = 0.09) Low back	
			C = Control (not further described)	33			
Luijsterburg (2005) Netherlands [52]	Non-RCT	Construction workers (bricklayer)	I = Devices for raised bricklaying	72	10 months	No (p = 0.65, p = 0.46, p = 0.95, p = 0.68, p = 0.68, p = 0.40) Low back, shoulder, hand-wrist	M
			C = Control (not further described)	130			
Risor (2017) Denmark [53]	Non-RCT	Nurses	I = Patient handling equipment, buying relevant equipment, training in its use	293	12 months	No (p > 0.05) Low-back, neck, shoulders, knees, and wrists	M
			C = Control (not further described)	201			
Sezgin (2018) Turkey [54]	Non-RCT	ICU nurses	I = An ergonomic risk management program based on the PRECEDE-PROCEED model	57	26 weeks	No (p = 0.633) All body parts/regions	M
			C = Control (not further described)	59			
Shabat (2005) Israel [55]	Non-RCT	Postal workers	I = Insoles	41	5 weeks	Yes (p < 0.05) Low back	M
					10 weeks	Yes (p < 0.05) Low back	
			C = Control (placebo insoles)	19			
**Participatory ergonomics**							
Brandt (2018) Denmark [57]	RCT	Construction workers	I = Participatory ergonomics: Reduce the number of events with excessive physical workload	32	3 months	No (p = 0.53) Arms, hands, knees, shoulder and back	H
					6 months	No (p = 0.59) Arms, hands, knees, shoulder and back	
			C = Control (handouts about MSD and lifting guidelines)	48			
Haukka (2008) Finland [58]	RCT	Kitchen workers	I = Participatory ergonomics: Identify strenuous work tasks and seek solutions for decreasing physical and mental workload	263	9–12 months	No (p > 0.05) 6 out of 7 body regions. Yes (p = 0.026) forearms/ hands	H
			C = Control (no visits and no trainings by researchers at these group)	241			
Jakobsen (2019) Denmark [59]	RCT	Healthcare workers	I = Participatory ergonomics: Improve the use of assistive devices in patient transfer	316	6 months	No (p = 0.868, p = 0.205, p = 0.117) Low back, shoulder, neck	H
					12 months	No (p > 0.05) Low back, shoulder, neck	
			C = Control (encouraged to continue with their normal working procedures including living up to standard OSH guidelines)	309			
Morken (2002) Norway [61]	RCT	Industry workers	I:1 = Participatory ergonomics training program with the operators and their supervisor	132	12 months	No (p > 0.05) All body parts/regions	M
			I:2 = Participatory ergonomics training program with operators only	135	12 months	No (p > 0.05) All body parts/regions	
			I:3 = Participatory ergonomics training program with managers and supervisors only	147	12 months	No (p > 0.05) All body parts/regions	
			C1 = Control (not receive any special attention or information)	423			
			C2 = Control (not receive any special attention or information)	1344			
Laing (2005) Canada [60]	Non-RCT	Manufacturing workers	I = Participatory ergonomics: Ergonomics change team implementing physical changes at the factory	44	10 months	No (p = 0.33, p = 0.52, p = 0.33, p = 0.96, p = 0.26, p = 0.50, p = 0.62, p = 0.05) Back, shoulder/upper arm forearm/hand and leg/lower limb	M
			C = Control (not further described)	39			
**Multifaceted**							
Chaleat-Valayer (2016) France [62]	RCT	Healthcare workers	I = Pain management education, exercise at workplace, exercise at home; booklet for self-management	171	18 months	No (p = 0.1417, p = 0.7002) Lumbar and radicular	H
			C = Control (usual care)	171			
Christensen (2011) Denmark [63]	RCT	Healthcare workers	I = Exercise; strength training, CBT, dietary	54	12 months	No (p = 0.452, p = 0.427, p = 0.476, p = 0.552) Neck, shoulder, upper back, lower back	H
			C = Control (a monthly two-hour oral lecture)	44			
Eriksen (2002) Norway [35]	RCT	Postal workers	I:2 = Exercise, information on stress, coping and practical examination (IHP)	165	12 weeks	No (p = 0.517) Head, neck, upper back and low back, arm, shoulder, and leg	H
			I:4 = Organizational intervention	199	12 weeks	No (p = 0.517) Head, neck, upper back and low back, arm, shoulder, and leg	
			C = Control (not further described)	344			
Ijzelenberg (2007) Netherlands [64]	RCT	Physical demanding workers (not specified)	I = Individually tailored education and training, immediate treatment of sub-acute LBP, ergonomic adjustment	258	12 months	No (p > 0.05) Low back, upper extremity	H
			C = Control (usual care)	231			
Jaromi (2018) Hungary [65]	RCT	Nurses	I = Back school program: Didactic education, spine-strengthening exercises and education in patient handling techniques	67	12 weeks	Yes (p < 0.001) Low back	H
			C = control (written lifestyle guidance)	70			
Jay (2015) Denmark [66]	RCT	Laboratory technicians	I = Physical, cognitive, and mindfulness group-based training	56	10 weeks	Yes (p < 0.0001) Neck, back, shoulder, elbow and hand	H
			C = Control (encouragement to participate in the company’s on-going health initiatives)	56			
Oude Hengel (2013) Netherlands [68]	RCT	Construction workers	I = Ergonomics, rest break, empowerment	171	3 months	No (p-value NA) Back, neck, shoulder, upper extremities, lower extremities	H
					6 months	No (p-value NA) Back, neck, shoulder, upper extremities, lower extremities	
					12 months	No (p-value NA) Back, neck, shoulder, upper extremities, lower extremities	
			C = Control (not further described)	122			
Peters (2018) USA [69]	RCT	Construction workers	I = Ergonomics + worksite health promotion	324	1 month	No (p = 0.252) All body parts/regions	H
					6 months	No (p = 0.683) All body parts/regions	
			C = Control (no intervention)	283			
Rasmussen (2015) Denmark [70]	RCT	Nurses	I = Participatory ergonomics, physical training, CBT (cross-over design)	594	12 weeks	Yes (p = < 0.0001) Low back	H
Roussel (2015) Belgium [71]	RCT	Hospital employees	I = Exercise, nutritional and psychological intervention, ergonomics	31	6 months	No (p > 0.05) Low back	H
			C = Control (not further described)	38			
Viester (2015) Netherlands [72]	RCT	Construction workers	I = Intervention mapping and coaching program	162	6 months	No (p > 0.05) Back, neck/shoulders, upper extremities, and lower extremities	H
					12 months	No (p > 0.05) Back, neck/shoulders, upper extremities and lower extremities	
			C = Control (usual care)	152			
Warming (2008) Denmark [49]	RCT	Nurses	I1 = Ergonomic; transfer technique instruction, physical exercise	50	12 months	No (p > 0.05) Low back	H
			C = Control (usual care)	76			
Tveito (2009) Norway [74]	RCT	Nurses	I = Exercise, ergonomic	19	9 months	No (p = 0.283, p = 0.220) Neck, back	M
			C = Control (no intervention)	21			
Kamioka (2011) Japan [67]	Non-RCT	Caregivers	I = Stretching exercise + ergonomic: learning	44	12 weeks	No (p = 0.653) Low back	H
			C = Control (not further described)	44			
Szeto (2010) Hong Kong [73]	Non-RCT	Nurses	I = Ergonomics training, exercise, education/theory (back school program)	14	8 weeks	No (p = 0.067) Shoulder, low back, neck, knee, elbow	M
			C = Control (no intervention)	12			
**Stress management**							
Eriksen (2002) Norway [35]	RCT	Postal workers	I:1 = Stress management training	162	12 weeks	No (p = 0.517) Head, neck, upper and lower back, arm, shoulder, and leg	H
			C = Control (not further described)	344			
Horneij (2001) Sweden [36]	RCT	Homecare workers	I:1 = Stress management	93	12 months	No (p = 0.057) Low back	H
					18 months	No (p = 0.063) Low back	
			C = Control (usual care)	99			
Jensen (2006) Denmark [45]	RCT	Healthcare workers	I:2 = Stress management	53	3 months	No (p = 0.64) Low back	H
					12 months	No (p = 0.85) Low back	
			C = Control (lessons on skincare, proper treatment of persons with diabetes, and asthma and safety procedures in chemicals handling)	49			
**Others**							
Jorgensen (2011) Denmark [42]	RCT	Cleaners	I:2 = CBT	99	12 months	No (p > 0.05) Neck, shoulder and low back	H
			C = Control (health care check, pulmonary test and aerobic capacity test)	100			
Sundstrup (2014) Denmark [77]	RCT	Slaughterhouses workers	I = Topical menthol	5	48 hours	Yes (p = 0.016, p = 0.027) Hand, forearm, elbow, wrist, arm	H
			C = Control (placebo gel)	5			
Faucett (2007) USA [75]	Non-RCT	Agriculture workers	I:1 = Rest breaks trial 1	30	3 days	Yes (p = 0.01) Mid/lower back and lower extremities	M
			I:2 = Rest breaks trial 2	16	3 days	Yes (p = 0.01) Mid/lower back and lower extremities	
			C = Control (only legally breaks)	36			
Wergeland (2003) Norway [76]	Non-RCT	Care institution workers	I = Reduced working hours	147	12 months	Yes (p = 0.034) Neck/shoulder. No (p = 0.320) Back	M
			C = Control (not further described)	286			

*QA* quality appraisal, *H* high quality study (> 85% of criteria met) and *M* medium quality study (50–85% of criteria met), *CBT* cognitive-behavioural therapy, *RCT *randomized controlled trial, *non-RCT *non-randomized controlled trial, *I* intervention group (if multiple intervention arms are present, *I *2 refers to intervention arm number 2 etc.), *C *control/comparison group

### Quality Appraisal

34 studies were classified as high quality (> 85% of criteria met), 20 studies were medium quality (50–85% of criteria met) and 9 studies were low quality (< 50% of criteria met). Only high and medium quality studies were eligible for the evidence synthesis whereas the summary table only describes these studies (see Table 4).

### Data Extraction

26 of the studies were published after 2012, 11 studies were published from 2008 to 2012, 11 studies were published from 2003 to 2007, and 6 studies were published from 1998 to 2002. 36 of the studies were RCTs and 18 studies were non-RCTs. Study designs under the umbrella “non-RCTs” included intervention studies, randomized intervention studies, cross-over intervention studies and clinical trials.

The majority of the 54 high and medium quality studies were published in Denmark (n = 18), with a further 6 performed in Norway, 5 in the Netherlands, 3 in the US, 3 in Italy, 3 in Japan, 2 in Sweden, 2 in Canada, 2 in France, 1 in Iran, 1 in Israel, 1 in Hungary, 1 in Belgium, 1 in Chile, 1 in Finland, 1 in Hong-Kong (China), 1 in Korea, 1 in Switzerland, and 1 in Turkey.

### Categorization into Intervention Domains and Categories

The interventions across the 54 studies were grouped into 5 intervention domains: physical exercise (n = 20), ergonomics (n = 13), participatory ergonomics (n = 5), multifaceted (n = 15) and stress management (n = 3). Within these domains, 3 interventional categories were further established, based on a practical and data-driven approach by the review-team: strength training (including strength training/resistance training alone or in combination with mobilization or stretching), aerobic training (including aerobic training/exercise and soccer) and stretching (stretching alone or in combination with warming-up or bodyweight exercises). Four studies did not match any of the intervention domains and are therefore discussed separately (see Table 4). Studies that encompass more than one intervention arm could be included more than one time under different intervention domains. Outcomes varied across the 54 included studies, but all studies included the outcome measures musculoskeletal pain, symptoms, prevalence or discomfort. The interventional effect from the included studies was classified as positive if the study reported positive results on these MSD-related outcome measures.

### Evidence Synthesis

Level of evidence from the 54 high and medium quality studies was synthesized on 5 broad intervention domains and 3 sub-categories within these domains. Level of evidence can be seen in Table 5. Importantly, no intervention domains were associated with "negative effects".

**Table 5:** Level of evidence and accompanying messages for stakeholders

Intervention category	Studies	Interventions	Consistency	Level of evidence	Message for stakeholders based on the scientific literature
Physical exercise	20	23	16 Effect (H = 8, M = 8); 7 No benefit (H = 6 M = 1)	Moderate (of a positive effect)	Practice consideration: Consider implementing physical exercise at the workplace for reducing MSD, especially if it is applicable to the work context
Strength training	9	9	9 Effect (H = 5, M = 4); 0 No benefit	Strong (of a positive effect)	Recommendation: Implementing strength training at the workplace can help reduce MSD among workers with physically demanding work
Aerobic training	5	5	3 Effect (H = 2, M = 1); 2 No benefit (H = 2, M = 0)	Limited	Not enough evidence from the scientific literature to guide current policies/practices
Stretching	3	4	2 Effect (H = 0, M = 2); 2 No benefit (H = 1, M = 1)	Mixed	Not enough evidence from the scientific literature to guide current policies/practices
Ergonomics	13	15	5 Effect (H = 2, M = 3); 10 No benefit (H = 5, M = 5)	Limited	Not enough evidence from the scientific literature to guide current policies/practices
Participatory ergonomics	5	7	7 No benefit (H = 3, M = 4)	Strong (for no benefit)	Not possible to make specific recommendations since the components of the participatory ergonomics interventions are so different
Multifaceted	15	16	3 Effect (H = 3, M = 0) 13 No benefit (H = 11, M = 2)	Strong (for no benefit)	Not possible to make specific recommendations since the components of the multifaceted interventions are so different
Stress management	3	3	0 Effect; 3 No benefit (H = 3, M = 0)	Strong (for no benefit)	Recommendation: Implementing a stress management intervention at the workplace seem to have no effect on reducing MSD among workers with physically demanding work
Others					
Rest breaks	1	2	2 Effect (M = 2)	Limited	Not enough evidence from the scientific literature to guide current policies/practices
Reduced working hours	1	1	1 Effect (M = 1)	Insufficient	
CBT	1	1	1 No benefit (H = 1)	Limited	
Topical analgesics	1	1	1 Effect (H = 1)	Limited	

### Physical Exercise

20 studies reporting on 23 different interventions were identified and grouped within the physical exercise domain [24–43]. 8 interventions form high-quality studies and 8 interventions from medium quality studies presented a positive effect of workplace exercise on MSD. Accordingly, there was moderate evidence of a positive effect of the domain of physical exercise. Thus, the present review results in the following message for stakeholders: Practice consideration: “Consider implementing physical exercise at the workplace for reducing MSD, especially if it is applicable to the work context”.

Within the domain of physical exercise, 9 studies reporting on 9 different interventions were identified and grouped within the strength training category [24–32]. 5 interventions form high-quality studies and 4 interventions from medium quality studies presented a positive effect of strength training at the workplace on MSD. There was strong evidence of a positive effect of strength training. This resulted in the following message for stakeholders: Recommendation. “Implementing strength training at the workplace can help reduce MSD among workers with physically demanding work”.

Within the domain of physical exercise, 5 studies reporting on 5 different interventions were identified and grouped within the aerobic training category [31, 33–36]. 2 interventions form high-quality studies and 1 intervention from a medium quality study presented a positive effect of aerobic training at the workplace on MSD. There was limited evidence of a positive effect of aerobic training. This resulted in the following message for stakeholders: “Not enough evidence from the scientific literature to guide current policies/practices”.

Within the domain of physical exercise, 3 studies reporting on 4 different interventions were identified and grouped within the stretching category [37–39]. 0 interventions form high-quality studies and 2 interventions from medium quality studies presented a positive effect of stretching at the workplace on MSD. There was mixed evidence of the effect of stretching. This resulted in the following message for stakeholders: “Not enough evidence from the scientific literature to guide current policies/practices”.

### Ergonomics

13 studies reporting on 15 different interventions were identified and grouped within the ergonomics domain [44–56]. 5 interventions from high-quality studies and 5 interventions from medium quality studies presented no effect of workplace ergonomics on MSD. There was limited evidence for no benefit for the domain of ergonomics. This resulted in the following message for stakeholders: “Not enough evidence from the scientific literature to guide current policies/practices”.

### Participatory Ergonomics

5 studies reporting on 7 different interventions were identified and grouped within the participatory ergonomics domain [57–61]. 3 interventions from high-quality studies and 4 interventions from medium quality studies presented no benefit of participatory ergonomics on MSD. There was strong evidence for no benefit for the domain of participatory ergonomics. Within this domain, the interventional components were so different, and our data-driven approach did not allow to further divide them into meaningful categories. This resulted in the following message for stakeholders: “Not possible to make specific recommendations since the components of the participatory ergonomics interventions are so different”.

### Multifaceted

15 studies reporting on 16 different interventions were identified and grouped within the multifaceted domain [35, 49, 62–74]. 11 interventions from high-quality studies and 2 interventions from medium quality studies presented no benefit of multifaceted workplace-interventions on MSD. There was strong evidence for no benefit for the domain of multifaceted interventions. Within this domain, the interventional components were so different, and our data-driven approach did not allow to further divide them into meaningful categories. This resulted in the following message for stakeholders: “Not possible to make specific recommendations since the components of the multifaceted interventions are so different”.

### Stress Management

3 studies reporting on 3 different interventions were identified and grouped within the stress management domain [35, 36, 45]. 3 interventions from high-quality studies presented no benefit of workplace stress management on MSD. There was strong evidence for no benefit for the domain of stress management. This resulted in the following message for stakeholders: Recommendation. “Implementing a stress–management intervention at the workplace seem to have no effect on reducing MSD among workers with physically demanding work.”

### Other Interventions

4 studies did not match any of the 5 intervention domains. Only one study for each of the following interventions was identified: rest breaks (medium quality study showing a positive effect) [75], reduced working hours (medium quality study showing a positive effect) [76], cognitive behavioral therapy (high-quality study showing no benefit) [42], topical analgesics (high-quality study showing a positive effect) [77]. This resulted in limited or insufficient evidence for each intervention type and the following messages for stakeholders: “Not enough evidence from the scientific literature to guide current policies/practices.”

## Discussion

54 suitable high or moderate quality studies were found reporting on the effect of 69 unique workplace interventions, serving as a solid foundation for the evidence synthesis and the subsequent recommendations for practitioners. There was moderate evidence of a positive effect of the domain of physical exercise at the workplace to reduce MSD among workers with physically demanding work. Within this domain, there was strong evidence of a positive effect of workplace strength training, where all 9 studies pointed in the same direction. There was limited evidence for the domain of ergonomics and thereby not enough evidence to guide current practices. There was strong evidence for no benefit for the domain of participatory ergonomics, multifaceted interventions, and stress management. The remaining single-domain intervention categories (rest breaks, reduced working hours, CBT, topical analgesics) only had one study each, and thereby not enough evidence to guide current practices. Importantly, no intervention domains were associated with "negative effects".

### Physical Exercise

16 of 23 interventions supported the domain of workplace physical exercise, resulting in a moderate level of evidence. Previous reviews have found evidence for the use of workplace exercise (not specified) for workers with upper limb, neck or back conditions/pain [17, 78] whereas others have not [14, 20, 79]. Kennedy et al. [20] found mixed evidence for exercise as an occupational health and safety intervention in the prevention of upper extremity MSDs among workers in general. This was however only based on four studies that all evaluated a somewhat similar exercise program that included a variety of activities such as strengthening, stretching, coordination, relaxation and/or stabilization exercises.

The many studies within the domain of physical exercise allowed for a further categorization into strength training, aerobic training, and stretching. All of the studies within the category of strength training showed an effect on MSD and therefore led to a strong level of evidence for a positive effect. These findings seemed to be consistent for both care/hospital workers and industry/manufacturing workers. A limited level of evidence was also found for aerobic training, whereas the studies on stretching showed mixed results. This is somewhat in line with previous reviews performed on the general working population or office workers. Van Eerd et al. [13] found strong evidence of resistance training, moderate evidence of stretching exercise programs, and limited evidence for a positive effect of aerobic training as workplace-based interventions in the prevention of upper extremity MSDs and symptoms. Further, Sihawong et al. [80] found strong evidence for the effectiveness of muscle strengthening and endurance exercises in treating neck pain among office workers. Skamagki et al. [16] found some consistency in their included studies, suggesting that high‐intensity strength training at the workplace can decrease pain and symptoms for employees who experience long‐term musculoskeletal disorders. However, at the time of that review, they also concluded that current research was limited. The present review underscores the importance of strength training as an effective intervention to reduce MSD among workers with physically demanding work.

In spite of this strong evidence, recent numbers from the Working Environment and Health study in Denmark shows that less than a third of Danish workers are offered physical exercise at the workplace [81]. Thus, future studies should investigate barriers to implementing physical exercise at the workplace rather than testing its effectiveness.

### Ergonomics

10 of 15 interventions showed no positive effect of workplace ergonomics on MSD leading to a limited level of evidence for no benefit for the domain of ergonomics: not enough evidence from the scientific literature to guide current policies/practices. Previous reviews on the general working population have both reported an effect, no effect and conflicting results of workplace ergonomics on MSD. Hoosain et al. [17] found positive effects for the use of ergonomic controls, ergonomic training and workstation adjustments, although these intervention categories had few high-quality studies. In opposition, Verbeek et al. [15] found no evidence available from RCTs for the effectiveness of manual material handling advice and training or manual material handling assistive devices for treating back pain. They concluded that more high-quality studies could further reduce the remaining uncertainty. Further, Verhagen et al. [79] found conflicting evidence concerning the effectiveness of ergonomic programs over no treatment in the treatment of work-related complaints of the arm, neck, or shoulder. In line with this, Van Eerd et al. [13] found mixed evidence for ergonomics training + workstation adjustment based on 8 studies and concluded that there is not enough evidence from the scientific literature to guide current policies/practices. They also reported moderate evidence of no benefit from workstation adjustment alone. As the ergonomic interventions were very heterogeneous, it should also be recognized that general conclusions about the effectiveness of workplace ergonomics should be done with care.

### Participatory Ergonomics

Participatory ergonomics means actively involving workers in developing and implementing workplace changes which will improve productivity and reduce risks to safety and health [82]. This is based on the assumption that workers are the experts,and, given appropriate knowledge, skills, tools, facilitation, resources, and encouragement, they are best placed to identify and analyze problems, and to develop and implement solutions which will be both effective in reducing injury risks and improving productivity and be acceptable to those affected [82, 83]. Despite these assumptions, we found that participatory ergonomics at the workplace had no effect on reducing MSD among workers with physically demanding work. Thus, all 7 interventions from high or medium quality studies showed no effect of participatory ergonomics, leading to a strong level of evidence for no benefit for this interventional domain. This is somewhat in disagreement with previous studies on the general working population. As an example, Rivilis et al. [84] found moderate evidence that participatory ergonomic interventions have a positive impact on MSD related symptoms. However, 3 of their 6 included studies were on sedentary workers (i.e. 2 studies on office workers and 1 study on garment workers) and their database search was performed until 2004. Further, Van Eerd et al. [13] found mixed evidence for low-intensity participatory ergonomics based on 4 studies on the general working population. Thus, sedentary workers of the general working population may have driven these positive effects reported in previous reviews. It has previously been suggested, that it may be more challenging to implement and study interventions among non-office workers [13]. Compared with office-work, the nature of work in workplaces with predominantly physically demanding work is obviously different and includes a great variety in work schedules, workstation design and work organization which could make it difficult to implement and conduct an evaluation. The present results on the effect of participatory ergonomics could therefore also reflect challenges in implementing such interventions at workplaces with physically demanding work. As the participatory ergonomics interventions were very heterogeneous, it should also be recognized that general conclusions about the effectiveness of participatory ergonomics should be done with care. A discussion on this can be seen in the “Methodological Considerations” below.

### Multifaceted Interventions

13 of 16 interventions from high or medium quality studies showed no effect of multifaceted workplace-interventions on MSD among workers with physically demanding employment. This resulted in a strong evidence level for no benefit for the domain of multifaceted interventions. In line with this, Van Hoof et al. [14] found very few low risk of bias RCTs and therefore concluded that there is no strong evidence for any intervention (including multidimensional interventions) in treating or preventing low back pain in nurses. Further, Dick et al. [85] found limited, but high quality, evidence that multidisciplinary rehabilitation for non-specific musculoskeletal arm pain, including both physical and psychosocial approaches, was beneficial for those workers absent from work for at least 4 weeks. In the present study, it was not possible to make recommendations to stakeholders since the components of the multifaceted interventions were so different. Lack of successful implementation could have contributed to the lack of effectiveness seen in some of the studies within this domain, which have been thought to be highly effective for reducing multifactorial outcomes such as MSD (further discussed below). Thus, it can not be ruled out, that there could have been more than 3 effective multifaceted interventions if implemented successfully. The 3 multifactorial interventions that were found to be effective in reducing MSD consisted of the following interventional components: (1) Spine Care for Nurses program consisting of didactic education, spine-strengthening exercises and education on safe patient handling techniques [65], (2) physical, cognitive, and mindfulness group-based training [66], and (3) participatory ergonomics, physical training, and cognitive-behavioural training [70]. Table 4 further describes the components of the multifactorial interventions that were found effective and not effective in the present review.

### Stress Management

3 of 3 interventions from high or medium quality studies showed no effect of stress management leading to a strong level of evidence for no benefit for this interventional domain. This is in line with previous reviews on upper limb and back pain among the general working population and among nurses [13, 14, 17]. For instance, Van Hoof et al. [14] found that stress management in isolation was not effective in nurses with and without low back pain and Van Eerd et al. [13] found moderate evidence for no effect of job stress management training for the prevention of upper extremity MSDs and symptoms.

### Practical Relevance

The prevention of MSDs at workplaces is a challenge and practitioners have therefore specifically asked for an evidence-based approach to better identify and implement effective interventions for employees with physically demanding work. In addition, implementing evidence-based initiatives at workplaces is a well-known challenge that may be due to the fact that existing knowledge is not conveyed clearly enough to the users, including the workplaces. Likewise, it is nearly an impossible task for OSH practitioners to find, read and synthesize relevant scientific literature on effective workplace solutions to reduce MSD. Employing the IWH review guidelines for the review provided us with the opportunity to develop relevant recommendations for practitioners. The involvement of relevant stakeholders in some of the review-steps has also maximized the practical relevance of the review and increased the opportunity for the evidence-based knowledge to reach relevant users [86–89]. By providing a solid and up-to-date evidence base with clear and understandable messages for practice, we hope that practitioners can be better suited to choosing the best solution to reduce MSD among employees with physically demanding work. Importantly, such messages and recommendations must not only be carefully crafted but also carefully delivered. The stakeholders will, therefore, be involved in both the development of practical tools—based on this review—and the delivery of both tools and messages to relevant workplaces. Notably, practitioners should also base the decision on what is relevant and applicable to their specific workplace context and take into consideration, that the results are based on the scientific literature, and not on the knowledge and know-how of practitioners and workplaces.

### Methodological Considerations

The perception of musculoskeletal pain and symptoms constitutes a complex interaction of both biological, psychological and social factors [4, 90]. MSD-related outcomes are therefore also complex measures that potentially can be affected by a multitude of factors. Further, the time-frame necessary before changes in MSD becomes apparent likely varies with the workplace intervention being delivered (i.e. intervention type) along with the population studied (e.g. intensity of MSD, functional consequence of MSD, duration of MSD). In the present study, the MSD-related outcomes included pain, symptoms, discomfort, or prevalence of MSD/pain. However, information was lacking in regard to the duration of MSD in the included studies. Even though duration is closely related to preventing MSD, which was not in focus for this review, it could have had an effect on the present results. In addition, some industries have itinerant workforces e.g. construction workers. Thus, seeing effects at longer time points is often complicated by not having the same workers that were exposed to the intervention. Thus, time-frames of outcome measurement could be an important factor for the effectiveness of workplace studies. For the present review, in case of several follow-up periods reported in the same study, data on MSD from the longest time-frame of outcome measurements were employed for the evidence synthesis (unless specifically stated in the study aim that a given follow-up time was the primary focus of the study). This definition could have introduced a certain amount of bias in the present reporting, especially if results on MSD appeared to vary between different time-frames. However, after inspecting the time-frames of outcome measurement, illustrated in Table 4, this was not an important factor that would change the overall evidence synthesis in the present review. Of all the included studies forming the evidence synthesis (Table 4), 15 studies (evaluating 17 different workplace interventions) had more than one follow-up measurement. Of these, only 3 studies (evaluating a total of 4 interventions) showed different results on MSD between the time-frames of outcome measurement: one study within the domain of ergonomics, and two studies within the domain of physical exercise, of which one was within the category of strength training. Deciding to use only the interventional effects of the shortest time-frames of outcome measurement would therefore not change the overall level of evidence for any of the interventional domains or categories.

Importantly, length to the latest time-frame of outcome measurement (i.e. study duration) varied between studies included in the different interventional domains. For instance, the average time to the latest outcome measurement was 19.6 weeks for interventions within the domain of physical exercise, whereas it was 42 weeks for ergonomics interventions, 41 weeks for participatory ergonomics interventions, and 31 weeks for multifaceted interventions. This could have influenced the present results and it also seems to highlight the need for investigating long-term effects of physical exercise at the workplace. Whether the present findings reflect a ceiling effect of the intervention effects after a short time-frame or a gradually diminishing adherence to the intervention occurs with time—and thereby limits further improvements—cannot be elucidated based on the information available in the included studies.

The interventional effect from the included studies was classified as positive if the study reported positive results on MSD-related outcomes such as pain, the prevalence of MSD/pain, symptoms, or discomfort. It should, however, be noted, that the effect was not necessarily based on the primary outcome results, but could also reflect secondary or tertiary outcome measures. As an example, in the included study by Brandt et al. [57] the primary outcome of the participatory ergonomics intervention was the number of events with excessive physical workload during a working day, while pain intensity in the last week (0–10 VAS-scale) was regarded as a secondary outcome measure. Thus, other study outcomes than those related to MSD could also be relevant and may have shown other results. Still, the review team and stakeholders decided that this was the best approach to answer the study's aim of investigating the effectiveness of workplace interventions to rehabilitate musculoskeletal disorders among workers with physically demanding employment. However, by focusing on these MSD related outcomes, the results do not necessarily say anything about the impact of MSD on disability level, activity limitations, and participation restrictions. The use of the International Classification of Functioning, Disability and Health model (ICF) [91] could, therefore, be a helpful tool in directing our attention to different aspects of functioning relevant to the workplace context rather than solely focusing on symptoms of MSD. Previous results from workplace interventions have also focused on other types of outcomes than pain, such as work ability and sick leave, which are more related to the employees functioning during daily work. Thus, future reviews could be inspired by the ICF framework and employ these specific aspects of functioning at a workplace level as effective measures of workplace interventions.

We found strong evidence for no benefit of participatory ergonomics and multifaceted interventions at the workplace. Importantly, within these domains (along with the domain of ergonomics), the interventional components were so different and our data-driven approach did not allow to further divide them into meaningful categories. There may have been many factors that could have contributed to the lack of effectiveness seen in some of these studies. Workplace interventions are complex and many factors can influence how the intervention was implemented, which in turn contributes to how effective they are. As mentioned above, the timeframe for outcome measurements could be an important factor because working conditions—although modifiable—can take a long time to modify due to the length of time required to implement new policies, practices or programs. Further, MSD is a complex outcome measure and it can take time to see any meaningful change. Importantly, every organization is different and interventions need to fit the company/workplace [92], which can be complicated by the fissured, multi-employer structure of some workplaces e.g. construction. Further, different industries are likely to have different working conditions and different interventions may, therefore, be more effective to MSD than others. For instance, multifaceted and participatory ergonomic intervention seem to be appropriate approaches for reducing the symptoms of MSD, even though we did not find evidence for this in the scientific literature [93]. Importantly, the results of such interventions do not only depend on the effectiveness of the effort itself, but also on the implementation strategy involving the planning and processing of the intervention so that it is integrated into the work organization and culture [93]. It can be very difficult to transfer a highly controlled and carefully planned intervention to practice since, in real life, management and not the researcher controls the implementation of workplace interventions and production systems and workflows are changeable [94, 95]. Lack of successful implementation could, therefore, have contributed to the lack of effectiveness seen in some of the studies in the present review. Thus, the conclusion of the present review regarding multifactorial and participatory interventions should be interpreted with caution.

As expected, substantial heterogeneity in the interventional outcome measures, study designs, and workplace contexts did not allow for the conduction of a meta-analysis. Specifically, outcome characteristics such as pain intensity, the prevalence of pain, symptoms, and discomfort were too broad to be matched or pooled and therefore lacked the comparability for a meaningful meta-analysis. This is also coherent with other reviews within the field of work-related interventions to reduce MSDs [13, 16, 17, 23]. Instead, we employed the pre-planned best evidence synthesis approach developed by IWH, with the opportunity to provide practitioners with the requested evidence-based approach to better identify and implement more relevant and effective workplace solutions. However, this approach does not consider sample size since small study populations count as much in the evidence synthesis as studies including a larger study sample.

### Strengths and Limitations

Including both RCTs and non-RCTs in the systematic review can both be considered a limitation and a strength. Including non-RCTs may downgrade the validity and strength of our systematic review and the risk of bias will become higher in the blinding and sequence generation domains. Therefore, we employ the IWH approach for the quality assessment and subsequent best evidence synthesis that are developed to handle other study designs than RCTs. Even though RCTs are considered the most powerful experimental design in clinical trials [96], solely including these may be too restrictive to understand effective workplace-based interventions where randomized and carefully controlled trials (RCTs) are not always possible. Hence, only including RCTs may exclude valuable information on workplace interventions to reduce MSDs among employees with physically demanding work. This is in line with various reviews within the field that have solely included RCTs and concluded that the current research is limited. Thus, the focus of the current review was to deliver the best evidence available for the practitioners and the employed best evidence synthesis was a transparent way of presenting this to our stakeholders. To maximize practical relevance we therefore correspondingly included non-RCTs and of the 54 high and moderate-quality studies included in the evidence synthesis, one third (i.e. 18 studies) were non-RCTs. We were, therefore, able to include valuable information that otherwise would have been excluded from the review if only RCTs were included.

Even though the IWH approach for the quality assessment and subsequent best evidence synthesis are developed to handle other study designs than RCTs, RCTs will, in general, have the possibility to obtain a higher quality score and have a higher impact on the level of evidence than non-RCTs. This is especially because some of the questions in the quality assessment form (Table 2) relates to the intervention allocation and the randomization process (the two questions within question number 3). Thus, information on RCT versus non-RCT was somewhat accounted for in the quality assessment using the IWH approach and therefore also in the subsequent evidence synthesis. In relation to this, we found that 88% of the included RCTs obtained a “high” quality score (i.e. > 85% of the maximum score) whereas this was the case for 22% of the non-RCTs. Further, within all the domains in the evidence synthesis, the results from the RCTs were in accordance with the results from the non-RCTs. However, within the intervention category of stretching, only non-RCTs existed, whereas only RCTs existed within the interventional domain of stress management.

A strength of the study is the involvement of relevant stakeholders in the review process, which ensured a high level of practical relevance. Stakeholder involvement in the design and search strategy phases introduced a more practical approach that allowed practitioners to share their knowledge and experience from practice along with their needs in regard to developing suitable workplace solutions.

Another strength is that authors were contacted to clarify whether potentially included studies were workplace-based if doubt about the location of the intervention existed based on the full-text reading. This led to the inclusion of several studies that otherwise would have been excluded for the review. Hence, this strengthens the foundation for the evidence synthesis.

Even though the included studies were from 19 different countries, differences in regard to the geographical distribution of studies clearly existed. Of the 54 studies included in the review, 27 studies were from Scandinavian countries (Denmark, Norway, Sweden, Finland) and only 13 studies were performed outside Europe. The present study results therefore mainly reflect the effectiveness of interventions in workplaces with physically demanding work in Europe and especially in the Scandinavian countries.

Publication bias cannot be ruled out from the present review. We attempted to be as inclusive as possible, as we expected that there weren’t that many eligible studies on workplace interventions to reduce MSD among employees with physically demanding work. Thus, studies with positive results were possibly more likely to be eligible for the present review. Even though the English language is generally perceived to be the universal language of science—also in regard to research within the field of work environment and health—only including these studies could have biased the present results by not representing all of the evidence available. Thus, the presence of a language restriction bias in the present study cannot be ruled out.

## Conclusions

The systematic search revealed 54 suitable high or moderate quality workplace-studies (36 RCTs and 18 non-RCTs) that focused on MSD among workers with physically demanding employment, which served as a solid foundation for the evidence synthesis and the subsequent recommendations for practitioners. The evidence synthesis recommends that implementing strength training at the workplace can reduce MSD among workers with physically demanding employment. In regard to workplace ergonomics, there was not enough evidence from the scientific literature to guide current practices. Based on the scientific literature, participatory ergonomics and multifaceted workplace interventions seem to have no beneficial effect on reducing MSD among this group of workers. As these interventional domains were very heterogeneous, it should also be recognized that general conclusions about their effectiveness should be done with care.

## Data Availability

The data that support the findings of this review will be available from the corresponding author upon reasonable request.

## References

[1] National Research Centre for the Working Environment. From the study: Work environment and health in Denmark; 2016. https://arbejdsmiljoidanmark.nfa.dk/

[2] Murray CJL, Barber RM, Foreman KJ, Ozgoren A, Abd-Allah F, GBD 2013 DALYs and HALE Collaborators (2015). Global, regional, and national disability-adjusted life years (DALYs) for 306 diseases and injuries and healthy life expectancy (HALE) for 188 countries, 1990-2013: quantifying the epidemiological transition. Lancet.

[3] Hartvigsen J, Hancock MJ, Kongsted A, Louw Q, Ferreira ML, Genevay S (2018). What low back pain is and why we need to pay attention. Lancet.

[4] Gatchel RJ, Peng YB, Peters ML, Fuchs PN, Turk DC (2007). The biopsychosocial approach to chronic pain: scientific advances and future directions. Psychol Bull.

[5] Pincus T, Kent P, Bronfort G, Loisel P, Pransky G, Hartvigsen J (2013). Twenty-five years with the biopsychosocial model of low back pain-is it time to celebrate? A report from the twelfth international forum for primary care research on low back pain. Spine.

[6] Shaw WS, van der Windt DA, Main CJ, Loisel P, Linton SJ, the “Decade of the Flags” Working Group (2009). Early patient screening and intervention to address individual-level occupational factors (“Blue Flags”) in Back disability. J Occup Rehabil..

[7] Wilkie R, Pransky G (2012). Improving work participation for adults with musculoskeletal conditions. Best Pract Res Clin Rheumatol.

[8] Andersen LL, Mortensen OS, Hansen JV, Burr H (2011). A prospective cohort study on severe pain as a risk factor for long-term sickness absence in blue- and white-collar workers. Occup Environ Med.

[9] Punnett L, Wegman DH (2004). Work-related musculoskeletal disorders: the epidemiologic evidence and the debate. J Electromyogr Kinesiol.

[10] Neupane S, Virtanen P, Leino-Arjas P, Miranda H, Siukola A, Nygård C-H (2013). Multi-site pain and working conditions as predictors of work ability in a 4-year follow-up among food industry employees. Eur J Pain.

[11] Natvig B, Eriksen W, Bruusgaard D (2002). Low back pain as a predictor of long-term work disability. Scand J Public Health.

[12] The Danish Working Environment Authority. Background note on the priority of musculoskeletal disorders; 2007.

[13] Van Eerd D, Munhall C, Irvin E, Rempel D, Brewer S, van der Beek AJ (2016). Effectiveness of workplace interventions in the prevention of upper extremity musculoskeletal disorders and symptoms: an update of the evidence. Occup Environ Med.

[14] Van Hoof W, O’Sullivan K, O’Keeffe M, Verschueren S, O’Sullivan P, Dankaerts W (2018). The efficacy of interventions for low back pain in nurses: a systematic review. Int J Nurs Stud.

[15] Verbeek JH, Martimo K-P, Karppinen J, Kuijer PPF, Viikari-Juntura E, Takala E-P (2011). Manual material handling advice and assistive devices for preventing and treating back pain in workers. Cochrane Database Syst Rev..

[16] Skamagki G, King A, Duncan M, Wåhlin C (2018). A systematic review on workplace interventions to manage chronic musculoskeletal conditions. Physiother Res Int.

[17] Hoosain M, de Klerk S, Burger M (2019). Workplace-based rehabilitation of upper limb conditions: a systematic review. J Occup Rehabil.

[18] Keown K, Van Eerd D, Irvin E (2008). Stakeholder engagement opportunities in systematic reviews: knowledge transfer for policy and practice. J Contin Educ Health Prof.

[19] Seeberg KGV, Andersen LL, Bengtsen E, Sundstrup E (2019). Effectiveness of workplace interventions in rehabilitating musculoskeletal disorders and preventing its consequences among workers with physical and sedentary employment: systematic review protocol. Syst Rev.

[20] Kennedy CA, Amick BC, Dennerlein JT, Brewer S, Catli S, Williams R (2010). Systematic review of the role of occupational health and safety interventions in the prevention of upper extremity musculoskeletal symptoms, signs, disorders, injuries, claims and lost time. J Occup Rehabil.

[21] Institute for Work and Health. Systematic review program—how we do systematic reviews: Institute for Work & Health [Internet]. IWH; 2018. https://www.iwh.on.ca/systematic-review-program/methods

[22] Slavin RE (1995). Best evidence synthesis: an intelligent alternative to meta-analysis. J Clin Epidemiol.

[23] Cullen KL, Irvin E, Collie A, Clay F, Gensby U, Jennings PA (2018). Effectiveness of workplace interventions in return-to-work for musculoskeletal, pain-related and mental health conditions: an update of the evidence and messages for practitioners. J Occup Rehabil.

[24] Jakobsen MD, Sundstrup E, Brandt M, Jay K, Aagaard P, Andersen LL (2015). Effect of workplace- versus home-based physical exercise on musculoskeletal pain among healthcare workers: a cluster randomized controlled trial. Scand J Work Environ Health.

[25] Rasotto C, Bergamin M, Sieverdes JC, Gobbo S, Alberton CL, Neunhaeuserer D (2015). A tailored workplace exercise program for women at risk for neck and upper limb musculoskeletal disorders: a randomized controlled trial. J Occup Environ Med.

[26] Sundstrup E, Jakobsen MD, Andersen CH, Jay K, Persson R, Aagaard P (2014). Effect of two contrasting interventions on upper limb chronic pain and disability: a randomized controlled trial. Pain Phys.

[27] Zebis MK, Andersen LL, Pedersen MT, Mortensen P, Andersen CH, Pedersen MM (2011). Implementation of neck/shoulder exercises for pain relief among industrial workers: a randomized controlled trial. BMC Musculoskelet Disord.

[28] Muñoz-Poblete C, Bascour-Sandoval C, Inostroza-Quiroz J, Solano-López R, Soto-Rodríguez F (2019). Effectiveness of workplace-based muscle resistance training exercise program in preventing musculoskeletal dysfunction of the upper limbs in manufacturing workers. J Occup Rehabil..

[29] Balaguier R, Madeleine P, Rose-Dulcina K, Vuillerme N (2017). Effects of a worksite supervised adapted physical activity program on trunk muscle endurance, flexibility, and pain sensitivity among vineyard workers. J Agromed.

[30] Jay K, Frisch D, Hansen K, Zebis MK, Andersen CH, Mortensen OS (2011). Kettlebell training for musculoskeletal and cardiovascular health: a randomized controlled trial. ScandJ Work EnvironHealth.

[31] Oldervoll LM, Rø M, Zwart JA, Svebak S (2001). Comparison of two physical exercise programs for the early intervention of pain in the neck, shoulders and lower back in female hospital staff. J Rehabil Med.

[32] Rasotto C, Bergamin M, Simonetti A, Maso S, Bartolucci GB, Ermolao A (2015). Tailored exercise program reduces symptoms of upper limb work-related musculoskeletal disorders in a group of metalworkers: a randomized controlled trial. Man Ther.

[33] Korshøj M, Birk Jørgensen M, Lidegaard M, Mortensen OS, Krustrup P, Holtermann A (2018). Decrease in musculoskeletal pain after 4 and 12 months of an aerobic exercise intervention: a worksite RCT among cleaners. Scand J Public Health.

[34] Barene S, Krustrup P, Holtermann A (2014). Effects of the workplace health promotion activities soccer and zumba on muscle pain, work ability and perceived physical exertion among female hospital employees. PLoS ONE.

[35] Eriksen HR, Ihlebaek C, Mikkelsen A, Grønningsaeter H, Sandal GM, Ursin H (2002). Improving subjective health at the worksite: a randomized controlled trial of stress management training, physical exercise and an integrated health programme. Occup Med (Lond).

[36] Horneij E, Hemborg B, Jensen I, Ekdahl C (2001). No significant differences between intervention programmes on neck, shoulder and low back pain: a prospective randomized study among home-care personnel. J Rehabil Med.

[37] Bertozzi L, Villafañe JH, Capra F, Reci M, Pillastrini P (2015). Effect of an exercise programme for the prevention of back and neck pain in poultry slaughterhouse workers: programme of prevention exercises in workers. Occup Ther Int.

[38] Holmström E, Ahlborg B (2005). Morning warming-up exercise—effects on musculoskeletal fitness in construction workers. Appl Ergon.

[39] Han H-I, Choi H-S, Shin W-S (2016). Effects of hamstring stretch with pelvic control on pain and work ability in standing workers. BMR.

[40] Andersen LN, Juul-Kristensen B, Roessler KK, Herborg LG, Sørensen TL, Søgaard K (2015). Efficacy of ‘Tailored Physical Activity’ on reducing sickness absence among health care workers: a 3-months randomised controlled trial. Man Ther.

[41] Gram B, Holtermann A, Bültmann U, Sjøgaard G, Søgaard K (2012). Does an exercise intervention improving aerobic capacity among construction workers also improve musculoskeletal pain, work ability, productivity, perceived physical exertion, and sick leave?: A randomized controlled trial. J Occup Environ Med.

[42] Jørgensen MB, Faber A, Hansen JV, Holtermann A, Søgaard K (2011). Effects on musculoskeletal pain, work ability and sickness absence in a 1-year randomised controlled trial among cleaners. BMC Public Health.

[43] Burger C, Schade V, Lindner C, Radlinger L, Elfering A (2012). Stochastic resonance training reduces musculoskeletal symptoms in metal manufacturing workers: a controlled preventive intervention study. Work.

[44] Hartvigsen J, Lauritzen S, Lings S, Lauritzen T (2005). Intensive education combined with low tech ergonomic intervention does not prevent low back pain in nurses. Occup Environ Med.

[45] Jensen LD, Gonge H, Jørs E, Ryom P, Foldspang A, Christensen M (2006). Prevention of low back pain in female eldercare workers: randomized controlled work site trial. Spine.

[46] Oleske DM, Lavender SA, Andersson GBJ, Kwasny MM (2007). Are back supports plus education more effective than education alone in promoting recovery from low back pain?: Results from a randomized clinical trial. Spine.

[47] Roelofs PDDM, Bierma-Zeinstra SMA, van Poppel MNM, Jellema P, Willemsen SP, van Tulder MW (2007). Lumbar supports to prevent recurrent low back pain among home care workers: a randomized trial. Ann Intern Med.

[48] Shojaei S, Tavafian SS, Jamshidi AR, Wagner J (2017). A multidisciplinary workplace intervention for chronic low back pain among nursing assistants in Iran. Asian Spine J.

[49] Warming S, EbbehØj NE, Wiese N, Larsen LH, Duckert J, TØnnesen H (2008). Little effect of transfer technique instruction and physical fitness training in reducing low back pain among nurses: a cluster randomised intervention study. Ergonomics.

[50] Hagiwara Y, Yabe Y, Yamada H, Watanabe T, Kanazawa K, Koide M (2017). Effects of a wearable type lumbosacral support for low back pain among hospital workers: a randomized controlled trial. J Occup Health.

[51] Iwakiri K, Sotoyama M, Takahashi M, Liu X, Koda S, Ichikawa K (2018). Effectiveness of re-education based on appropriate care methods using welfare equipment on the prevention of low back pain among care workers: a 1.5 year follow-up study. Ind Health..

[52] Luijsterburg PAJ, Bongers PM, de Vroome EMM (2005). A new bricklayers’ method for use in the construction industry. Scand J Work Environ Health.

[53] Risør BW, Casper SD, Andersen LL, Sørensen J (2017). A multi-component patient-handling intervention improves attitudes and behaviors for safe patient handling and reduces aggression experienced by nursing staff: a controlled before-after study. Appl Ergon.

[54] Sezgin D, Esin MN (2018). Effects of a PRECEDE-PROCEED model based ergonomic risk management programme to reduce musculoskeletal symptoms of ICU nurses. Intensive Crit Care Nurs.

[55] Shabat S, Gefen T, Nyska M, Folman Y, Gepstein R (2005). The effect of insoles on the incidence and severity of low back pain among workers whose job involves long-distance walking. Eur Spine J.

[56] Yassi A, Cooper JE, Tate RB, Gerlach S, Muir M, Trottier J (2001). A randomized controlled trial to prevent patient lift and transfer injuries of health care workers. Spine.

[57] Brandt M, Madeleine P, Samani A, Ajslev JZ, Jakobsen MD, Sundstrup E (2018). Effects of a participatory ergonomics intervention with wearable technical measurements of physical workload in the construction industry: cluster randomized controlled trial. J Med Internet Res.

[58] Haukka E, Leino-Arjas P, Viikari-Juntura E, Takala E-P, Malmivaara A, Hopsu L (2008). A randomised controlled trial on whether a participatory ergonomics intervention could prevent musculoskeletal disorders. Occup Environ Med.

[59] Jakobsen MD, Aust B, Kines P, Madeleine P, Andersen LL (2019). Participatory organizational intervention for improved use of assistive devices in patient transfer: a single-blinded cluster randomized controlled trial. Scand J Work Environ Health.

[60] Laing A, Frazer M, Cole D, Kerr M, Wells R, Norman R (2005). Study of the effectiveness of a participatory ergonomics intervention in reducing worker pain severity through physical exposure pathways. Ergonomics.

[61] Morken T, Moen B, Riise T, Hauge SHV, Holien S, Langedrag A (2002). Effects of a training program to improve musculoskeletal health among industrial workers—effects of supervisors role in the intervention. Int J Ind Ergon.

[62] Chaléat-Valayer E, Denis A, Abelin-Genevois K, Zelmar A, Siani-Trebern F, Touzet S (2016). Long-term effectiveness of an educational and physical intervention for preventing low-back pain recurrence: a randomized controlled trial. Scand J Work Environ Health.

[63] Christensen JR, Faber A, Ekner D, Overgaard K, Holtermann A, Søgaard K (2011). Diet, physical exercise and cognitive behavioral training as a combined workplace based intervention to reduce body weight and increase physical capacity in health care workers—a randomized controlled trial. BMC Public Health.

[64] IJzelenberg H, Meerding W-J, Burdorf A (2007). Effectiveness of a back pain prevention program: a cluster randomized controlled trial in an occupational setting. Spine..

[65] Járomi M, Kukla A, Szilágyi B, Simon-Ugron Á, Bobály VK, Makai A (2018). Back School programme for nurses has reduced low back pain levels: a randomised controlled trial. J Clin Nurs.

[66] Jay K, Brandt M, Hansen K, Sundstrup E, Jakobsen MD, Schraefel MC (2015). Effect of individually tailored biopsychosocial workplace interventions on chronic musculoskeletal pain and stress among laboratory technicians: randomized controlled trial. Pain Phys.

[67] Kamioka H, Okuizumi H, Okada S, Takahashi R, Handa S, Kitayuguchi J (2011). Effectiveness of intervention for low back pain in female caregivers in nursing homes: a pilot trial based on multicenter randomization. Environ Health Prev Med.

[68] Oude Hengel KM, Blatter BM, van der Molen HF, Bongers PM, van der Beek AJ (2013). The effectiveness of a construction worksite prevention program on work ability, health, and sick leave: results from a cluster randomized controlled trial. Scand J Work Environ Health.

[69] Peters S, Grant M, Rodgers J, Manjourides J, Okechukwu C, Dennerlein J (2018). A cluster randomized controlled trial of a Total Worker Health® intervention on commercial construction sites. IJERPH.

[70] Rasmussen CDN, Holtermann A, Bay H, Søgaard K, Birk JM (2015). A multifaceted workplace intervention for low back pain in nursesʼ aides: a pragmatic stepped wedge cluster randomised controlled trial. Pain.

[71] Roussel NA, Kos D, Demeure I, Heyrman A, Clerck MD, Zinzen E (2015). Effect of a multidisciplinary program for the prevention of low back pain in hospital employees: a randomized controlled trial. BMR.

[72] Viester L, Verhagen EALM, Bongers PM, van der Beek AJ (2015). The effect of a health promotion intervention for construction workers on work-related outcomes: results from a randomized controlled trial. Int Arch Occup Environ Health.

[73] Szeto GPY, Law KY, Lee E, Lau T, Chan SY, Law S-W (2010). Multifaceted ergonomic intervention programme for community nurses: pilot study. J Adv Nurs.

[74] Tveito TH, Eriksen HR (2009). Integrated health programme: a workplace randomized controlled trial. J Adv Nurs.

[75] Faucett J, Meyers J, Miles J, Janowitz I, Fathallah F (2007). Rest break interventions in stoop labor tasks. Appl Ergon.

[76] Wergeland EL, Veiersted B, Ingre M, Olsson B, Akerstedt T, Bjørnskau T (2003). A shorter workday as a means of reducing the occurrence of musculoskeletal disorders. Scand J Work Environ Health.

[77] Sundstrup E, Jakobsen MD, Brandt M, Jay K, Colado JC, Wang Y (2014). Acute effect of topical menthol on chronic pain in slaughterhouse workers with carpal tunnel syndrome: triple-blind, randomized placebo-controlled trial. Rehabil Res Pract.

[78] Boocock MG, McNair PJ, Larmer PJ, Armstrong B, Collier J, Simmonds M (2007). Interventions for the prevention and management of neck/upper extremity musculoskeletal conditions: a systematic review. Occup Environ Med.

[79] Verhagen AP, Karels C, Bierma-Zeinstra SMA, Feleus A, Dahaghin S, Burdorf A (2007). Exercise proves effective in a systematic review of work-related complaints of the arm, neck, or shoulder. J Clin Epidemiol.

[80] Sihawong R, Janwantanakul P, Sitthipornvorakul E, Pensri P (2011). Exercise therapy for office workers with nonspecific neck pain: a systematic review. J Manip Physiol Ther.

[81] Bach E, Andersen LL, Bjørner JB. Work environment and health in Denmark; 2018. https://nfa.dk/da/nyt/nyheder/2011/samlet-rapport-om-arbejdsmiljoe-og-helbred-i-danmark-2010.

[82] Burgess-Limerick R (2018). Participatory ergonomics: evidence and implementation lessons. Appl Ergon.

[83] Brown O, Stanton N, Hedge A, Brookhuis K, Salas E, Hendrick H (2005). Participatory ergonomics. Handbook of human factors and ergonomics methods.

[84] Rivilis I, Van Eerd D, Cullen K, Cole DC, Irvin E, Tyson J (2008). Effectiveness of participatory ergonomic interventions on health outcomes: a systematic review. Appl Ergon.

[85] Dick FD, Graveling RA, Munro W, Walker-Bone K, on behalf of the Guideline Development Group (2011). Workplace management of upper limb disorders: a systematic review. Occup Med..

[86] Diderichsen F, Nygaard E, Bonde A. Collaboration between research and practice in the field of prevention. The Danish Health Authority; 2009.

[87] Carpenter WR, Meyer A-M, Wu Y, Qaqish B, Sanoff HK, Goldberg RM (2012). Translating research into practice: the role of provider-based research networks in the diffusion of an evidence-based colon cancer treatment innovation. Med Care.

[88] Grimshaw JM, Eccles MP, Lavis JN, Hill SJ, Squires JE (2012). Knowledge translation of research findings. Implement Sci..

[89] Bumbarger BK, Campbell EM (2012). A state agency-university partnership for translational research and the dissemination of evidence-based prevention and intervention. Adm Policy Ment Health Ment Health Serv Res.

[90] Noonan J, Wagner SL (2010). A biopsychosocial perspective on the management of work-related musculoskeletal disorders. AAOHN J..

[91] World Health Organization. International classification of functioning, disability and health: ICF. Geneva: World Health Organization; 2001.

[92] Peters SE, Nielsen KM, Nagler EM, Revette AC, Madden J, Sorensen G (2019). Ensuring organization-intervention fit for a participatory organizational intervention to improve food service workers’ health and wellbeing: Workplace organizational health study. J Occup Environ Med..

[93] Roquelaure Y (2008). Workplace intervention and musculoskeletal disorders: the need to develop research on implementation strategy. Occup Environ Med.

[94] Takala E-P. Ergonomic interventions and prevention—a need for better understanding of implementation. Scand J Work Environ Health [Internet]. 2018 [cited 2020 Jan 10]; https://www.sjweh.fi/show_abstract.php?abstract_id=3710 10.5271/sjweh.3710 29355290

[95] Winkel J, Westgaard RH (2019). Development and implementation of interventions managing work-related musculoskeletal disorders: inadequacy of prevalent research framework and future opportunities. Scand J Work Environ Health.

[96] Stolberg HO, Norman G, Trop I (2004). Randomized controlled trials. Am J Roentgenol.

